# Margin Assessment in Soft Tissue Sarcomas: Review of the Literature

**DOI:** 10.3390/cancers13071687

**Published:** 2021-04-02

**Authors:** Andrea Sambri, Emilia Caldari, Michele Fiore, Riccardo Zucchini, Claudio Giannini, Maria Giulia Pirini, Paolo Spinnato, Alberta Cappelli, Davide Maria Donati, Massimiliano De Paolis

**Affiliations:** 1 Alma Mater Studiorum, University of Bologna, 40126 Bologna, Italy; davide.donati@ior.it; 2 IRCCS Policlinico di Sant’Orsola, 40138 Bologna, Italy; emilia.caldari@aosp.bo.it (E.C.); mariagiulia.pirini@aosp.bo.it (M.G.P.); alberta.cappelli@aosp.bo.it (A.C.); massimiliano.depaolis@aosp.bo.it (M.D.P.); 3 IRCCS Istituto Ortopedico Rizzoli, 40136 Bologna, Italy; michele.fiore@ior.it (M.F.); riccardo.zucchini@ior.it (R.Z.); claudio.giannini@ior.it (C.G.); paolo.spinnato@ior.it (P.S.)

**Keywords:** margins, soft tissue sarcoma, classification, infiltrative, local recurrence

## Abstract

**Simple Summary:**

Many classifications to assess margins status for soft tissue sarcomas are reported in the literature. Most of the series are heterogeneous and variable in size, making it difficult to compare results from study to study. Thus, which is the best way to assess margins in order to predict the risk of local recurrence is still debated. The aim of this narrative review is to provide a comprehensive assessment of the literature on margins, and to highlight the need for a uniform description of the margin status for patients with soft tissue sarcomas (STS).

**Abstract:**

Adequacy of margins must take into consideration both the resection margin width (quantity) and anatomic barrier (quality). There are several classification schemes for reporting surgical resection margin status for soft tissue sarcomas (STS). Most of the studies regarding treatment outcomes in STS included all histologic grades and histological subtypes, which include infiltrative and non-infiltrative subtypes and are very heterogeneous in terms of both histologic characteristics and treatment modalities (adjuvant treatments or not). This lack of consistency makes it difficult to compare results from study to study. Therefore, there is a great need for evidence-based standardization concerning the width of resection margins. The aim of this narrative review is to provide a comprehensive assessment of the literature on margins, and to highlight the need for a uniform description of the margin status for patients with STS. Patient cases should be discussed at multidisciplinary tumor boards and treatments should be individualized to clinical and demographic characteristics, which must include also a deep knowledge of specific histotypes behaviors, particularly infiltrative ones.

## 1. Introduction

Surgical excision with a broad margin of the surrounding normal tissue is the mainstay of treatment for localized soft tissue sarcomas (STS) [1,2], in order to minimize the risk of local recurrence (LR). Current guidelines recommend complete resection of STS with a negative surgical margin, without a specific recommendation for the width of that margin or a standard definition of a negative margin [3]. Due to their rarity and heterogeneity, most studies regarding treatment outcomes in STS included all histologic grades and histologies (infiltrative and non-infiltrative subtypes) [4], leading to general outcomes, which are intricate to use in a clinical setting.

An adequate margin must take into consideration both the margin width (quantity) and the type of anatomic barrier (quality) [5]. There are several classification schemes for reporting surgical resection margin status for STS (Table 1). The Musculoskeletal Tumor Society (MSTS) classification originally described by Enneking [6] defined radical margins (extracompartmental en-bloc excision) and wide margins (intracompartmental en-bloc with a cuff of normal tissue). However, this classification did not detailed how to assess margins for intracompartmental resections (marginal and intralesional excision). Over the years, different residual tumor classification systems have been established. The American Joint Committee on Cancer (AJCC)—R classification categorizes margins as negative (R0), microscopically positive (R1), or grossly positive (R2) [7]. In 2002, the Union Against Cancer (UICC) proposed a R + 1 mm classification, which requires 1 mm of normal tissue between tumor and margin to define a negative margin (R0) [8,9], thus resulting in more resections being considered microscopically positive (R1). The Toronto Margin Context Classification (TMCC) outlined four categories: negative margins (R0 according to the R classification), unplanned positive margins, a planned close but with an ultimately positive microscopic margin along a critical structure, and a positive margin after a tumor bed re-excision in patients treated initially with inadequate surgery elsewhere (the whoops procedure) [8,10]. A solely positive or negative status of a margin, however, gives no insight into the difference between clear but close margins and broader margins. Thus, international guidelines typically recommend reporting tumor clearance (metric distance of surgical margins) [2]. However, the minimum margin distance necessary to reduce the risk of LR of high-grade STS remains undefined.

If the tumor is resected with a broad margin, a variety of tissues may exist between the resection margin and tumor. Anatomic factors such as the tissue composition of the margin may impact the appropriateness of margin [6,10], with dense regular connective tissue assumed to provide a more robust anatomic barrier to tumor cell infiltration. However, the significance of margin quality has been investigated rarely, so the efficacy of these structures as a barrier is still debated. Rydholm and Rooser [11], considering an intact muscle as a distinct anatomical compartment, subclassified wide Enneking margins as wide-S (subcutaneous) when a subcutaneous tumor was excised with a cuff of subcutaneous tissue and deep fascia, wide-F (fascia) when a deep tumor was excised with an intact envelope of uninvolved fascia, and wide-AM (areolar tissue and muscle). This subclassification provided LR risk stratification, with a 10% 5-year rate of LR with wide-S and wide-F margins and 30% with a wide-AM margin. Kawaguchi et al. [12] proposed a method of margins assessment based on a combination of margin quantity and quality. Margins were classified as curative, adequate, or inadequate depending on the width and quality of the tissue comprising the margin. Inadequate wide margins did not ensure local control even with adjuvant radiotherapy in high-grade sarcomas but were sufficient for low-grade tumors.

The aim of this narrative review is to provide a comprehensive assessment of the literature on margins, and to highlight the need for a uniform description of the margin status for patients with STS.

## 2. Margins and Imaging

Modern imaging facilitates successful functional limb salvage, often with margins that would be classified as marginal in the Enneking system [13]. Precisely this distinction is important in the light of sparing as much tissue as possible for preferable functional outcomes [14]. Magnetic resonance imaging (MRI) is the imaging tool most sensitive and accurate to assess margins in STS. On conventional MRI, there are several recognized features related to margins infiltration and subsequent increased risk of LR after surgical excision. Above all, the most important feature on baseline MRI is an infiltrative grown pattern. This pattern is generally characterized by irregular tumor surface and invasion in the surrounding tissue; the infiltrative pattern is usually defined as focal (<25% of tumor circumference) or diffuse (>25% of tumor circumference) [15]. In series focused on the MRI evaluation of surrounding tissue in STS, the recognition of the infiltrative grown pattern was found to be significantly associated with satellite tumorous cells at surgical specimen histological analysis [16,17,18,19,20]. Some infiltrative STS subtypes (myxofibrosarcoma and undifferentiated pleomorphic sarcoma) may present a specific focal-infiltrative pattern, the so-called ‘tail sign’: a curvilinear tumor spread from the principal mass, usually along the fascial plane. This sign is associated with increased risk of LR after excision; thus, its recognition in pre-operative studies is fundamental for the complete and safe excision of all tumoral components with broader margins of resection needed [21].

Peritumoral enhancement after Gadolinium administration is a well-recognized prognostic factor in STS associated with patients’ worst local control. In a series of 130 STS patients, Crombé et al. [22] observed that peritumoral enhancement was the most significant MRI feature associated with histologically high-grade sarcomas. The presence of this feature can be considered a direct manifestation of surrounding tissue infiltration in most cases and its recognition should also suggest ampler margins of resection.

In order to reduce the risk of LR, intraoperative ultrasound was recently reported as a very useful tool for ameliorating excision of STS [23].

Recent technical development of imaging analyses and the increasing request for evidence-based medicine, led to a greater use of quantitative imaging. One of the most promising tool is radiomics: it is a quantitative tool applicable to imaging, which aims at enhancing the existing data by means of advanced mathematical analysis. Through mathematical extraction of the spatial distribution of signal intensities and pixel interrelationships, this tool quantifies textural data by using analysis methods from the field of artificial intelligence [24]. Radiomics has already been proven to predict margins invasion in a quantitative manner in several malignancies and it seems to be of great potential even for STS [25,26,27]. Radiomics applied on MRI images can offer valuable quantitative information regarding tumor shape that may help in the assessment of margins infiltration [27]. However, future studies focused on radiomics and STS margins infiltration are needed to assess the potential use of this technology [28].

## 3. Margins Assessment

A recent consensus practice guideline remarked that “no available evidence-based data addressed how to adequately assess margins” [3]. Pathologists assess surgical resection margins by submitting more sections from margins that are close or concerning on gross inspection. However, careful gross assessment of surgical resection margins can be misleading. Therefore, microscopic examination of six to eight perpendicular sections in total from all margins <2 cm in width is a practical compromise and a reasonable recommendation [29].

Goldstein et al. [30] examined the shrinkage of the resected specimens in colon cancer, observing that sample shrank by 57% of the in vivo length after formalin fixation. Given that this contraction also occurs in STS, the microscopic 2 mm margin, for example, could be equivalent to 4–5 mm. In addition, there is also contraction of normal muscle or adipose tissue around the tumor just after excision. Therefore, when considering a possible shrinkage down to the microscopic 2 mm margin, it could be safer to plan for an at least 1 cm margin in the surgical field in order to minimize the risk of local failure for STS.

Only a few reports have shown the diagnostic accuracy of intraoperative frozen-section diagnoses for STS [31,32,33]. However, the role of frozen-section for margins assessment in STS has never been investigated. Frozen-section diagnoses exhibited low diagnostic accuracies for adipocytic tumors and fibroblastic/myofibroblastic tumors [34].

Nevertheless, intraoperative assessment of surgical margins is critical to ensuring residual tumor does not remain in a patient. Therefore, fluorescence-guided cancer surgery has been proposed also in STS. Fu et al. [35] and Matsubara et al. [36] investigated the role of acridine orange to highlight residual disease after the tumor was surgically removed. Acridine orange has an additional photodynamic effect which may help in sterilizing residual disease [36]. Another fluorescent agent (Indocyanine green) was recently investigated in animal models of synovial sarcoma [37].

## 4. Comparison between Classifications

Many series in the literature report local recurrence rates after surgical treatment of STS according to different classifications (Table 2, Table 3, Table 4, Table 5 and Table 6). However, only a few recent studies directly analyzed which classification is most accurate in predicting LR of STS. 

Cates et al. [38] compared different margin classification schemes (R classification, MSTS, or margin distance method) and they observed that none of them was clearly superior to the others. However, they reported that, given the increased specificity and positive predictive value of a binary positive versus negative reporting system, the dichotomized R system and MSTS classifications appear sufficient in this clinical setting. Gundle et al. [39] by direct comparison of three schemes (R, R+1 and TMCC classifications) concluded that the traditional R classification best determined the risk of LR, but TMCC provides additional stratification of positive margins that may aid in the surgical planning. Kainhofer et al. [40] compared different classifications of resection margins (R and R+1 classifications) on LR and found out that margin status according to both classifications was a significant prognostic factor for LR. However, they observed that a R0 margin determined by the UICC classification is a better discriminator for LR than R0 resections according to the R classification. A higher percentage of positive resection margins naturally results by applying the stricter definition of the UICC classification [40]. This indicates that R1 resections as defined by the UICC-classification pick out more patients with high risk of LR than R1 resections according to the R-classification would do.

## 5. Margins Width

Only a few studies reported that margins have no impact on LR [43,54], whereas most of the series observed that tumor resection with a gross positive margin are accompanied by unacceptably high rates of LR [41,51,55,56]. However, a solely positive or negative status of a margin, as clear but close margins and broader margins should not be considered equal in their risk to the patient [8]. The real question is what constitutes an “optimal” margin? How ample a surgical margin is needed to ensure the lowest risk of LR while still preserving function? However, this question has never been satisfactorily addressed in the literature [29].

Adequate margin has been described in several ways, ranging from adjectives like a radical, wide, or close [6] until negative (>0 mm) margin [52,57], up to 3 cm [53,58], or even 5 cm [53]. Ahmad et al. [52] and Harati et al. [59] reported that achieving a negative margin is essential for optimizing local control and survival, but the absolute quantitative width of the negative margin does not influence outcome. According to King et al. [60], the incidence of LR was similar in patients with less than 1 mm margins and greater than 1 mm margins. On the other hand, many series reported a 1 mm width as a good margin. Kainhofer et al. [40] concluded that patients with a resection margin of <1 mm have the same local control as those with positive resection margin [58]. Gundle et al. [39] observed that in the setting of radiation treated patients, a negative but <1 mm margin may be adequate. Similarly, Cates et al. [38] demonstrated that close but negative margins (<1 mm from tumor or MSTS marginal resection margins) appear adequate after neoadjuvant therapy, even if the risk of LR is slightly decreased with broader resection margins (≥2 mm or MSTS wide/radical margins). In the study of Dickinson et al. [61], margins of <1 mm or contaminated margins proved to be worse, but margins of 1–4 mm, 5–9 mm, and 10–19 mm showed about the same rate of LR. Other series reported that margins as close as 2 mm are sufficient [46,62]. Other authors suggested that margins should be at least 10 mm to ameliorate local control [63,64]. Nevertheless, it is virtually impossible to achieve a margin width of 10 mm or more for the vast majority of large, high-grade, deep-seated STS; thus, defining an optimal margin width of 10 mm is not clinically feasible in most patients.

Two different series reported that a minimum 5 mm margin is adequate if no adjuvant radiation therapy (RT) is administered, but it can be reduced to 1 mm with post-operative RT [29,65]. Sadoski et al. [66], as well as many other series [10,13,44,47,52,60,66], suggested that patients who have microscopic positive margins have lower rates of LR as long as the surgical excision is coupled with adjuvant RT. On the other hand, another series [44] observed that positive margins were associated with lower local control despite the utilization of adjuvant RT, suggesting negative margins remains a primary objective for all treatment options. These findings suggest that although microscopic disease left behind at the time of surgery may in some cases be sterilized by RT, this may be incomplete, and LR is still a significant risk under such circumstances. Therefore, achieving an optimal, negative surgical margin is of paramount importance.

Unexpected positive margins not along critical structures but, instead, affecting the soft tissues surrounding the excised STS may occur because of surgical error or extension of tumor beyond what is evident on cross-sectional imaging, and they have the highest risk of LR [39]. However, microscopically positive margins can be also the consequence of close dissection for the preservation of major vessels, nerves, or bone [8]. While unexpected positive margins showed the worst outcome for LR, “planned” positive margins along critical structures is acceptable in particular in patients who receive preoperative RT [45,50].

## 6. Margins in Specific STS Histotypes

Specific STS subtypes such as myxofibrosarcoma (MFS) and undifferentiated pleomorphic sarcoma (UPS) frequently present with an infiltrative growth pattern [19,20,42,48,49,67]. They have an increased likelihood of LR irrespective of the surgical margins [68]. Therefore, it is crucial to analyze the entities separately and to assess the prognostic significance of treatment-related factors for every histologic subset specifically. Regarding the effects of margins status in UPS, Dineen et al. [69] found an association between R0 margins and local control, whereas Le Doussal et al. [70] observed that only R2-resections were found to be of prognostic significance while microscopic margins did not alter the outcome. Iwata et al. [42] concluded that a resection margin of 2 cm away from the end of the radiological tumor infiltration would be the ideal margin because the vast majority of cases could achieve broad histological in infiltrative sarcomas resection seems to be overstated. Nevertheless, such a broader resection can increase the demand for plastic reconstruction surgery, risk of wound complication, and functional deterioration.

Dadrass et al. [71] in a homogeneous series of MFS, found an association of LR with positive margin resection. Interestingly, the rate of LR in negative margin resections was relatively high compared to other STS subtypes. Sambri et al. [16] observed no correlation between margin adequacy and LR in MFS. This highlights that specific infiltrative STS can highly recur even if excised with wide margins. Although negative margins may be achieved during resection, the irregular histologic pattern of MFS can still reveal microscopically positive margins on histopathology, more often than would be expected in other histotypes [71]. Fujiwara et al. [72] demonstrated that in the case of MFS and UPS, neither the R-classification nor the R1-classification were able to stratify the risk of LR in patients with negative margins, indicating that these classification systems are not sufficiently sensitive to stratify what constitutes an adequate margin of resection for infiltrative STS. Nonetheless, the authors observed that a margin in excess of 10 mm was associated with the lowest LR risk. In addition, LR risk in patients with margins < 10 mm with fascia/periosteum barrier was found to have an equivalent risk of LR compared to resection margin greater than 10 mm with any margin quality [4]. Therefore, a margin composed of fascia may be equivalent to a metric margin of at least 10 mm. Goertz et al. [73] observed that only the quality of surgical margins, but not the negative margin width had a prognostic influence. Moreover, it must be considered that preoperative RT may not have an effect on these infiltrative STS subtypes [16,67].

Well-differentiated liposarcoma is known to rarely recur. However, most of the series reported data on tumor grade rather than on the specific histology. Fujiwara et al. [74] in a homogeneous series on low-grade STS observed that excellent local control was achieved with margins >2 mm, even though negative margins provided only 10% LR. The authors concluded that the role of margins is more important than RT in local control for low-grade STS. Similarly, Sampo et al. [53] reported that LR risk in patients with low-grade STS was 21.4% at 5 years follow-up in patients with positive margins and 9.8% in those with negative margins. Kawaguchi et al. [12,51] also analyzed the adequacy of margin width in low-grade sarcomas, but their cohort included a mixture of bone and soft tissue sarcomas.

## 7. Conclusions

Most of the studies regarding treatment outcomes in STS included all histologic grades and histologies (infiltrative and non-infiltrative subtypes) [4,75,76] and are very heterogeneous in terms of both histologic characteristics and treatment modalities (adjuvant treatments or not). This lack of consistency as well as very variable sample size between studies, makes it difficult to compare study results. Therefore, there is a great need for evidence-based standardization concerning the width of resection margins. None of the reported classifications is completely reliable for predicting the risk of recurrence.

Patient cases should be discussed at multidisciplinary tumor boards and treatments should be individualized to clinical and demographic characteristics, which must include also a deep knowledge of specific histotypes behavior [77]. Additional treatment should, therefore, be considered for all tumors that are resected with close negative margins. In patients with small superficial tumors, this may best be accomplished by re-excision. In patients who have already undergone the amplest possible excision, or in whom complete and accurate re-excision would be difficult, adjuvant radiotherapy should be considered.

A careful surgical planning and the administration of neoadjuvant RT have evolved to allow closer and even positive margins around critical structures to preserve critical structures and thereby maximize patient function [8,10,56,60]. A fair discussion with the patient regarding the increased risk of LR versus the increased morbidity and functional impairment of a broader resection is warranted. Moreover, these patients should be recognized to be at special risk for local failure, and should be considered as prime candidates for new treatments designed to enhance local control.

## Figures and Tables

**Table 1:** Classifications of margins in soft tissue sarcomas.

Classification	MSTS [6]	AJCC [7]	UICC [9]	Tumor Clearance [2]	TMCC [10]	Margin Quality [6]
Description	Radical: all normal tissue involved anatomic compartment excised en-bloc	R0: tumor does not reach intact barrier or resection margins	R0: resection margin >1 mm	Metric distance from edge of tumor to inked surgical resection margin	Negative margins: tumor does not reach intact barrier or resection margins	Anatomic factors (the tissue composition of the margin)
-	Wide: histologically non-reactive normal tissue at margin	R1: Microscopic tumor contamination of margins or resection alongside pseudocapsule	R1: Resection margin < 1 mm	-	Unplanned positive margins	-
-	Marginal: pseudocapsule present at margin	R2: Macroscopic tumor contamination	R2: Macroscopic tumor contamination	-	Planned close but with an ultimately positive microscopic margin along a critical structure	-
-	Intralesional: Tumor present at margin	-	-	-	Positive margin after a tumor bed re-excision in patients treated initially with inadequate surgery elsewhere (“whoops procedure”)	-

AJCC: American Joint Committee on Cancer (R classification); MSTS: Muscoloskeletal Tumor Society; UICC: Union Against cancer; TMCC: Toronto Margin Context Classification.

**Table 2:** Review of the literature. Studies reporting margins based on MSTS classification.

Study	Margins	Sample Size	Histotypes	Tumor Grade	Median Follow Up (Months)	LR Rate	Other Prognostic Factors for LR?	Margin Indipendent Prognostic Factor for LR?	Homogeneous Cohort
Vodanovich et al. [37]	Radical/Wide Marginal/Intralesional	266	Infiltrative STS	High grade	85	13.0% 36.9%	yes	Age	yes
Cates et al. [28]	Radical/Wide Marginal Intralesional	166	All histotypes	High grade	51	12.0% 24.0% 50.0%	yes	-	no
Cates et al. [26]	Radical/Wide Marginal/Intralesional	166	All histotypes	High grade	49	11.0% (no RT) 3.0% (with RT) 100.0% (no RT) 35.0% (with RT)	yes	RT	no
Willeumier et al. [41]	Intralesional Marginal Wide	127	Infiltrative STS	High grade	71	50.0% 30.0% 8.0%	yes	Tumor size	yes

LR: local recurrence; RT: radiotherapy.

**Table 3:** Review of the literature. Studies reporting margins based on AJCC classification.

Study	Margins	Sample Size	Histotypes	Tumor Grade	Median Follow Up (Months)	LR Rate	Margin Indipendent Prognostic Factor for LR?	Other Prognostic Factors for LR?	Homogeneous Cohort
Gundle et al. [29]	R0% R1 R2	2217	All Histotypes	All grades	65	6.0% 17.0% 38.0%	yes	Grade 3 Depth RT	no
Stojadinovic et al. [42]	R0% R1	2084	All histotypes	High- low grade	50	15.2% 28.0%	yes	Tumor size Histologic grade Retroperitoneal location Fibrosarcoma histologic subtype	no
Harati et al. [43]	R0 R1	643	All histotypes	All grades	64	32.9% 63.9%	yes	Age Histological grade Myxofibrosarcoma Adjuvant RT	no
Ahmad et al. [37]	R0% R1	382	All histotypes	All grades	82	7.0% 17.0%	yes	RT	no
Bilgeri et al. [35]	R0% R1	305	All Histotypes	Grade 2–3	60	17.0% 34.0%	yes	RT	no
Kainhofer et al. [31]	R0% R1	265	All histotypes	All grades	-	16.5% 36.7%	yes	Adjuvant ChT	no
Le Doussal et al. [44]	R0% R1	216	Infiltrative STS	All grades	42	37.0%	no	RT	no
Goertz et al. [45]	R0% R1/R2	192	Infiltrative STS	All grades	61	24.3% 50.9%	yes	Adjuvant RT	yes
Cates et al. [28]	R0% <1 mm ≥2 mm R1-R2	166	All histotypes	High grade	51	24.0% 12.0% 50.0%	yes	-	no
Cates et al. [26]	R0% R1	166	All histotypes	High grade	49	30.0% (no RT) 17.0% (with RT) 17.0% (with RT)	yes	RT	no
Iwata et al. [46]	R0% R1 R2	145	All histotypes	Grade 2–3	48	5.0% 21.0% 15.0%	yes	Histological infiltration	no
Sambri et al. [15]	R0% R1	129	Infiltrative STS	Grade 3	35	18.0% 29.4%	no	-	yes
Dadrass et al. [47]	R0% R1/R2	42	Infiltrative STS	All grades	-	13.0% 85.0%	yes	-	yes

LR: local recurrence; RT: radiotherapy; ChT: chemotherapy.

**Table 4:** Review of the literature. Studies reporting margins based on UICC classification.

Study	Margins	Sample Size	Histotypes	Tumor Grade	Median Follow Up (Months)	LR Rate	Margin Indipendent Prognostic Factor for LR?	Other Prognostic Factors for LR?	Homogeneous Cohort
Gundle et al. [29]	R0% R1 R2	2217	All Histotypes	All grades	65	6.0% 10.0% 38.0%	yes	Grade 3 Depth RT	no
Kainhofer et al. [31]	R0% R1	265	All histotypes	All grades	-	12.0% 57.9%	yes	Adjuvant ChT Tumor Depth	no
Fuiiwara et al. [48]	R0% R1 R2	109	All Histotypes	Grade 1	67	7.0% 14.0% 54.0%	yes	Tumor Depth	no

LR: local recurrence; RT: radiotherapy; ChT: chemotherapy.

**Table 5:** Review of the literature. Studies reporting margins based on TMCC classification.

Study	Margins	Sample Size	Histotypes	Tumor Grade	Median Follow Up (Months)	LR Rate	Margin Indipendent Prognostic Factor for LR?	Other Prognostic Factors for LR?	Homogeneous Cohort
Gundle et al. [29]	Negative margins Critical structure positive margins Tumor bed resection positive margins Unexpected positive margins	2217	All Histotypes	All grades	65	6.0% 10.0% 18.0% 28.0%	yes	Grade 3 Depth RT	no
O’Donnell et al. [8]	Negative margins Critical structure positive margins Tumor bed resection positive margins Unexpected positive margins	1371	All histotypes	All grades	62	3.0% 14.6% 21.1% 36.6%	yes	-	no
Kawaguchi et al. [34]	Positive margin <1 mm 1–4 mm 5 mm	837	All histotypes	High- low grade	-	79.0% 40.0% 11.0% 10.0%	yes	-	no

LR: Local recurrence; RT: radiotherapy.

**Table 6:** Review of the literature. Studies reporting margins based on width margin classification.

Study	Margins	Sample Size	Histotypes	Tumor Grade	Median Follow Up (Months)	LR Rate	Margin Indipendent Prognostic Factor for LR?	Other Prognostic Factors for LR?	Homogeneous Cohort
Harati et al. [43]	Positive margin ≤1 mm >1 mm/≤5 mm >5 mm	643	All histotypes	All grades	64	63.9% 30.9% 31.3% 32.4%	yes	Age Histological grade Myxofibrosarcoma subtype Adjuvant RT	no
Gannon et al. [49]	Positive margin ≤1 mm >1 mm	514	All histotypes	All grades	19.2	9.0% 4.2% 9.3%	No	RT	no
Fujiwara et al. [50]	Positive margin 0.1–9.9 mm ≥10 mm	278	Infiltrative STS	Grade 2–3	60	22.0% 13.0% 3.0%	yes	Margin quality	yes
Ahmad et al. [38]	Positive margin ≤1 mm >1 mm/≤5 mm >5 mm	382	All histotypes	All grades	82	17.0% 10.0% 7.0% 0%	yes	RT	no
Bilgeri et al. [35]	Positive margin ≤1 mm >1 mm/≤5 mm >5 mm	305	All histotypes	Grade 2–3	60	34.0% 30.0% 13.0% 0%	yes	RT	no
Dickinson et al. [51]	Positive margin <1 mm 1–4 mm 5–9 mm 10–19 mm ≥20 mm	279	All histotypes	-	32	22.0% 17.0% 4.0% 5.0% 9.0% 0%	yes	-	no
Sampo et al. [39]	≥1 cm ≥2 cm ≥2.5 cm	270	All histotypes	High- low grade	79	16.7% 14.1% 10.8%	yes	RT	no
Goertz et al. [45]	Positive margin ≤1 mm >1 mm/≤5 mm >5 mm	192	Infiltrative STS	All grades	61	50.9% 21.8% 13.1% 25.0%	yes	Adjuvant RT	yes
Liu et al. [52]	Positive margin 1–4 mm 5–9 mm 10–19 mm 20–29 mm ≥30 mm	181	All histotypes	All grades	43	71.0% 42.0% 42.0% 3.0% 9.0% 0%	yes	Tumor Depth	no
Cates et al. [26]	<3 mm >3 mm	166	All histotypes	High grade	49	57.0% (no RT) 31% (with RT) 9.0% (no RT) 4.0% (with RT)	yes	RT	no
Sadoski et al. [53]	Positive margin ≤1 mm >1 mm	132	All histotypes	All grades	-	18.0% 6.0% 3.0%	yes	-	no
Willeumier et al. [41]	Positive margin 0–2 mm >2 mm	127	Infiltrative STS	High grade	71	50.0% 30.0% 8.0%	yes	Tumor size	yes
King et al. [54]	<1 mm 1–5 mm >5 mm	117	All histotypes	All grades	44	4.4% 2.6% 3.8%	no	-	no
Mcknee et al. [55]	Positive margin 1–2 mm 3–9 mm ≥10 mm	111	All histotypes	High- low grade	45	42.0% 39.0% 31.0% 16.0%	yes	Tumor size	no
Tang et al. [49]	<2 mm >2 mm Positive margin Re-excision Radiotherapy No treatment	73	All histotypes	High- low grade	52	45.0% 11.0% 20.0% 57.1% 100.0%	yes	-	no

LR: local recurrence; RT: radiotherapy.
